# ^11^C-L-methyl methionine dynamic PET/CT of skeletal muscle: response to protein supplementation compared to L-[ring ^13^C_6_] phenylalanine infusion with serial muscle biopsy

**DOI:** 10.1007/s12149-017-1157-4

**Published:** 2017-03-04

**Authors:** Emily J. Arentson-Lantz, Isra H. Saeed, Lynda A. Frassetto, Umesh Masharani, Roy J. Harnish, Youngho Seo, Henry F. VanBrocklin, Randall A. Hawkins, Carina Mari-Aparici, Miguel H. Pampaloni, James Slater, Douglas Paddon-Jones, Thomas F. Lang

**Affiliations:** 1 Department of Nutrition and Metabolism and Center for Recovery, Physical Activity and Nutrition (CeRPAN), University of Texas Medical Branch, Galveston, 77573 USA; 2 Department of Radiology and Biomedical Imaging, University of California, San Francisco, Box 0946, San Francisco, CA 94143-0946 USA; 3 Department of Medicine, University of California, San Francisco, San Francisco, CA 94143 USA

**Keywords:** PET/CT, Human, Muscle protein synthesis, FSR

## Abstract

**Objective:**

The objective of this study was to determine if clinical dynamic PET/CT imaging with ^11^C-L-methyl-methionine (^11^C-MET) in healthy older women can provide an estimate of tissue-level post-absorptive and post-prandial skeletal muscle protein synthesis that is consistent with the more traditional method of calculating fractional synthesis rate (FSR) of muscle protein synthesis from skeletal muscle biopsies obtained during an infusion of L-[ring ^13^C_6_] phenylalanine (^13^C_6_-Phe).

**Methods:**

Healthy older women (73 ± 5 years) completed both dynamic PET/CT imaging with ^11^C-MET and a stable isotope infusion of ^13^C_6_-Phe with biopsies to measure the skeletal muscle protein synthetic response to 25 g of a whey protein supplement. Graphical estimation of the Patlak coefficient K_i_ from analysis of the dynamic PET/CT images was employed as a measure of incorporation of 11 C-MET in the mid-thigh muscle bundle.

**Results:**

Post-prandial values [mean ± standard error of the mean (SEM)] were higher than post-absorptive values for both K_i_ (0.0095 ± 0.001 vs. 0.00785 ± 0.001 min^−1^, *p* < 0.05) and FSR (0.083 ± 0.008 vs. 0.049 ± 0.006%/h, *p* < 0.001) in response to the whey protein supplement. The percent increase in K_i_ and FSR in response to the whey protein supplement was significantly correlated (*r* = 0.79, *p* = 0.015).

**Conclusions:**

Dynamic PET/CT imaging with ^11^C-MET provides an estimate of the post-prandial anabolic response that is consistent with a traditional, invasive stable isotope, and muscle biopsy approach. These results support the potential future use of ^11^C-MET imaging as a non-invasive method for assessing conditions affecting skeletal muscle protein synthesis.

## Introduction

Muscle-wasting conditions place a significant burden on the public health system and are a threat to independence and quality of life [1, 2]. Our ability to develop pharmacologic, nutritional, or exercise based countermeasures to muscle/function loss is intrinsically tied to the availability of quantitative methods to characterize their effects on the physical and biochemical properties of skeletal muscle tissue.

In research environments, the measurement of acute localized skeletal muscle protein fractional synthesis rate (FSR) is traditionally used to quantify the response to an anabolic stimulus [3, 4]. This moderately invasive procedure includes a stable isotope infusion (e.g., L- [ring-^13^C_6_] phenylalanine (^13^C-Phe)), peripheral venous blood sampling, and serial needle muscle biopsies, typically from the vastus lateralis. The stable isotope, 3-pool modeling technique provides an estimate of protein synthesis and breakdown, but requires femoral venous and arterial catheterization [5]. While these methodologies continue to be used successfully in a variety of clinical research environments [6, 7], they may be contraindicated for clinically vulnerable populations. Thus, a technology to rapidly and non-invasively evaluate skeletal muscle protein anabolism would have significant impact on the assessment of muscle-wasting and associated countermeasures in people unable to tolerate prolonged, more invasive studies.

In the late 1990s, Fischman and colleagues [8] introduced dynamic positron emission tomography (PET) imaging of skeletal muscle [8, 9]. They combined an infusion of ^11^C-L-Methyl-Methionine (^11^C-MET) with three compartment kinetic modeling to provide a minimally invasive, in vivo estimate of skeletal muscle protein synthesis (MPS) in potentially any muscle bed, at any anatomic site. An animal study demonstrated that uptake of ^11^C-MET in skeletal muscle is highly correlated with its stable isotope analog (^13^C-L-Methyl-Methionine) [9], and basal (i.e., fasted and resting) kinetic model parameters have successfully been evaluated in adult humans [8]. However, until recently, there were no data evaluating the ability of PET imaging to quantify MPS changes in response to known anabolic stimuli. In 2012, Harnish et al. showed that ^11^C-MET responded to the anabolic stimulus of exercise-induced muscle fatigue [10]. In this study of unilateral knee extension and flexion, ^11^C-MET uptake was increased 1-h post-exercise in the exercised compared to the control leg. This was reflected in a side-to-side difference in the Patlak Coefficient (*K*_*i*_), a measure of forward extraction of the imaging agent into the skeletal muscle protein compartment [10].

The studies referenced above support the potential to use non-invasive imaging methodology to assess the anabolic response of skeletal muscle to clinically relevant nutritional and pharmacologic interventions used to treat skeletal muscle atrophy in patient groups such as sarcopenic elderly, and patients with muscle-wasting conditions such as chronic kidney disease, HIV, or diabetes. However, to be considered for evaluation of MPS in these populations, it is important to show that measures derived from imaging correlate with information coming from gold standard techniques such as ^13^C-Phe infusion/biopsy. Although PET/CT and stable isotope infusion/biopsies use different tracers (^11^C-MET and ^13^C-Phe) to measure K_i_ and FSR, respectively, both methodologies effectively target the biological process of protein synthesis by quantifying the incorporation of an isotopically labeled amino acid into the skeletal muscle tissue. Therefore, the goal of this study was to determine feasibility of clinical dynamic PET/CT imaging with ^11^C-MET in healthy older women to provide an estimate of tissue-level post-absorptive and post-prandial skeletal MPS that is consistent with FSR calculated from skeletal muscle biopsies obtained during an infusion of ^13^C-Phe. We chose to use older women for this current study, because they tend to be at high risk for sarcopenia [11], but are anecdotally less likely to volunteer for biopsy studies and are more difficult to biopsy because of lower lean mass, making them a population that would benefit from the development of non-invasive methodology.

## Materials and methods

### Subjects

Healthy, community-dwelling, post-menopausal, tracer naïve women aged 66–80 years (average age 73 ± 5 years; *n* = 13) were recruited from the greater San Francisco Bay Area. Study protocols and procedures were conducted in accordance with the Declaration of Helsinki and reviewed and approved by the Institutional Review Board of the University of California San Francisco (UCSF). After providing written informed consent, volunteers were medically screened in the UCSF Clinical Research Center (CRC). Subjects were excluded if they had been diagnosed with any musculoskeletal or metabolic disease, including diabetes, uncontrolled hypertension, or autoimmune disease, as previously described [12].

### Procedures

**Fig. 1:**
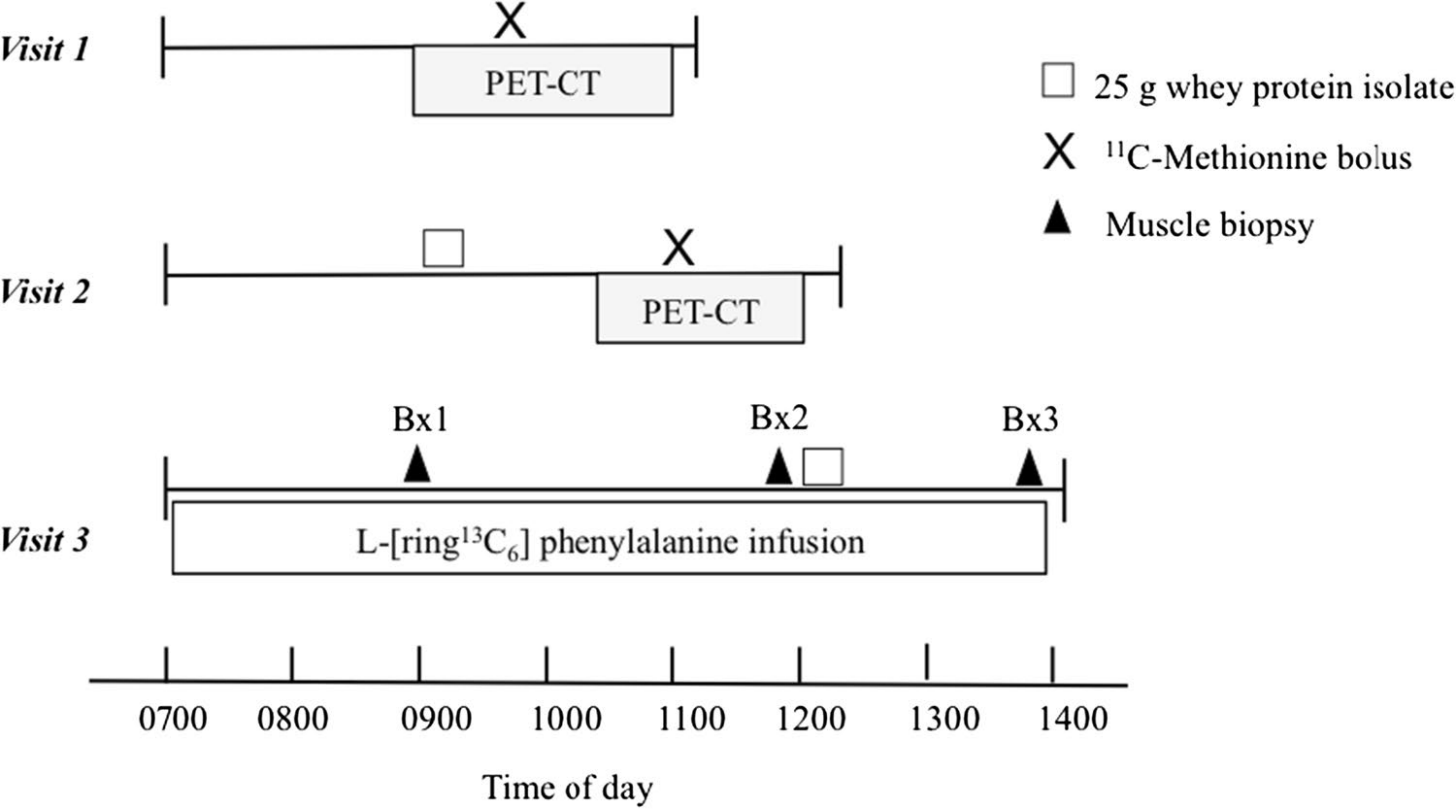
Study time-lines. *Visit 1* Assessment of post-absorptive MPS using PET/CT with a ^11^C-MET bolus. *Visit 2* Assessment of post-prandial MPS using PET/CT. *Visit 3* Assessment of post-absorptive and post-prandial FSR using ^13^C-Phe and muscle biopsies. All visits were separated by 1–2 weeks

All subjects completed three study visits in sequential order, with each visit separated by 1–2 weeks. The protocols to measure changes in K_i_ (PET/CT) required two imaging visits, one in which the subjects were imaged after an overnight fast (water only) and one in which the subjects arrived in the fasting state and then consumed a 25-g whey protein isolate supplement (BiPro, Davisco, Le Sueur, MN) 1 h prior to imaging. The FSR (muscle biopsy) protocol involved an infusion of ^13^C-Phe during which biopsies were obtained immediately before and 2 h after ingestion of the whey protein supplement. The timing of the imaging and infusion biopsy protocols is summarized in Fig. 1.

### PET/CT image acquisition

For the imaging visits, subjects reported to the China Basin Outpatient Imaging Center at the UCSF Department of Radiology and Biomedical Imaging. All images were acquired using a Discovery VCT PET/CT camera (GE Healthcare, Chicago, IL, USA). Subject was oriented in the scanner in the supine position, with legs extended into the scanner aperture and feet straight up and taped together. An anteroposterior scout view depicted the lower body anatomy from the iliac crest to the tibial plateau and was employed to determine a CT scanning interval from the mid-thigh to a point roughly 2-cm superior to the distal femoral condyle. The dynamic PET axial field of view, which extended 14.5 cm along the table axis (the fixed width of the PET detector array), was centered in the CT volume. Based on the CT scan volume defined from the scout view, the subject was imaged with a helical CT scan (120 kVp, 300 mAs, 3.75-mm sections, 512^2^ matrix, and standard reconstruction kernel). After the CT image was acquired, the subject was injected with a bolus of 462-MBq (range 407–481 MBq) ^11^C-MET solution, resulting in an approximately 2-mSv effective dose to the subjects. Beginning concurrently with the ^11^C-MET bolus injection, the dynamic PET data were acquired using a sequence of 27 temporally contiguous frames over the course of 60 min. The time frames were of variable durations, acquired in the following sequence: [15 × 10 s, 5 × 30 s, 4 × 5 min, 2 × 10 min, 1 × 15 min]. Images were reconstructed into voxels of dimensions 1.95 × 1.95 × 3.27 mm^3^ in 256 × 256 × 47 matrices via a 3D ordered subset expectation maximization algorithm (28 subsets, two iterations) with CT-based attenuation correction provided by the scanner manufacturer.

### PET/CT image analysis

To determine the rate of incorporation of ^11^C-MET into bound skeletal muscle protein, we employed the Patlak graphical analysis [13], which can be used to determine the fractional extraction rate *K*_*i*_ from plasma into a peripheral compartment (bound protein). The method assumes that the tissue compartment is in rapid equilibrium with the plasma and bound peripheral compartments, and that release from the bound compartment is not observed in the timescale of the study. To derive *K*_*i*_ from the dynamic images, the ratio *C*_T_(*t*)/*C*_A_(*t*) is plotted against ∫*C*_A_(*t*)d*t*/*C*_A_(*t*), integrated between *t* = 0 and *t* = 60 min, where *C*_A_(*t*) is the concentration as a function of time *t* of the tracer ^11^C-MET in the femoral artery as represented by the image-derived input function (IDIF, see below), and *C*_T_(*t*) is the concentration as a function of time measured in the tissue activity curve (TAC). When the system achieves equilibrium between the three compartments, the Patlak plot becomes linear and the Patlak slope K_i_ can be derived from fitting that linear component. In a previous study [10], we reported a high correlation (*r* = 0.92) between *K*_*i*_ and the MPS rate estimated by Fishman et al. [8] from kinetic analysis using a three compartment model of dynamic PET data. Because of the robustness and simplicity of the Patlak *K*_*i*_, we utilized it as a measure of image-derived MPS in this study.

**Fig. 2:**
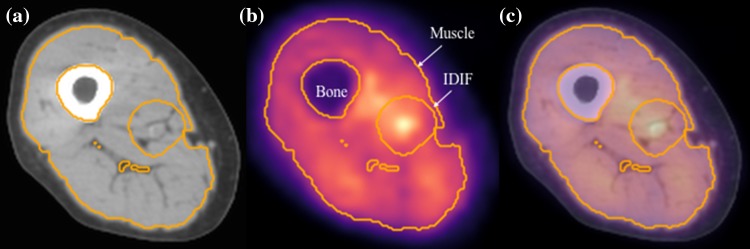
Images from Subject 004. CT (**a**) and PET (**b**) images corresponding to central mid-thigh cross section of thigh volume. The fused PET/CT image is shown in (**c**). The tissue activity curve (TAC) was computed from the region encompassing the total thigh muscle volume, but excluding bone and the region used to determine the image-derived input function (IDIF). These regions are superimposed in gold pixels on all three images

To estimate the contribution of arterial blood flow to the skeletal muscle uptake of ^11^C-MET as a function of time *C*_A_(*t*), we computed an image-derived arterial input function (IDIF) from region of interest analysis around the femoral artery as previously described [14]. The implementation of the IDIF for analysis of the dynamic images in our study was described by Harnish et al. [10]. To quantify the uptake of ^11^C-MET in skeletal muscle, ^11^C-MET concentrations were measured from regions of interest (ROIs) determined automatically from the CT scans using threshold-driven region growing. The distal femoral condyle and the lesser trochanter, two bony landmarks visible on the anteroposterior scout view, were used to establish the inferior and superior limits of the set of axial slices included in the TAC ROI. The inferior limit of the ROI occurred in the axial slice closest to 36% of the distance from the femoral condyle to the lesser trochanter, and the superior limit occurred in the slice closest to 44% of that distance. To avoid contamination of the tissue region by arterial counts, a region surrounding the artery, roughly the extent of the outer radius of the annular region used to subtract tissue background counts from the arterial signal, was excluded from the skeletal muscle tissue ROI. Representative PET/CT images and the region used for derivation of the arterial input function are shown in Fig. 2.

### Stable isotope and biopsy methodology

1–2 weeks after the completion of the first imaging visit, volunteers were admitted to the UCSF-CRC. An 18-gauge polyethylene catheter (Insyte-W; Becton Dickinson, Sandy, UT) was inserted into the forearm vein of each arm, one for blood sampling, and one for stable isotope infusion. After baseline blood sampling to measure background phenylalanine enrichment, a primed (2 μmol/kg), constant infusion (0.06 μmol/kg/min) of L- [ring-^13^C_6_] phenylalanine was started and continued throughout the study (Fig. 1). A 5-mm Bergstrom needle was used to take a muscle biopsy from the vastus lateralis under local anesthesia following a standardized technique [4]. Subjects ingested 25 g of whey protein mixed with 250 mL of water immediately following the second biopsy. This high-quality protein has been shown to provide a rapid, near maximal increase in skeletal muscle protein synthesis [15]. Peripheral venous blood samples and the final muscle biopsy were obtained according to the schedule outlined in Fig. 1.

### Processing of blood and skeletal muscle samples

Venous blood samples were immediately mixed with 1 mL ice-cold sulfosalicylic acid and centrifuged for 20 min (3000 rpm at 4 °C). The supernatant was removed and stored at -80 °C. Muscle samples were blotted and rinsed with saline, flash frozen in liquid nitrogen, and stored at −80 °C. All muscle and blood samples were sent to the University of Texas Medical Branch for analysis. Phenylalanine enrichment of muscle proteins as well as plasma were determined as previously described [7, 12].

Mixed muscle fractional synthesis rate was calculated by measuring the direct incorporation of ^13^C_6_-Phe into protein using the precursor-product model [7]:

$$\text{FSR}=~\frac{[{{E}_{\it {B}}}\left( t2 \right)-{{E}_{\it B}}\left( t1 \right)]}{[t2-t1]}/\frac{[{{E}_{\it {p}}}\left( t1 \right)+{{E}_{\it {p}}}\left( t2 \right)]}{2}\ \times ~\ 60~\ \times \ ~100$$

where *E*_B_ is the protein-bound phenylalanine enrichment across two sequential biopsies taken at time *T*1 and *T*2, respectively, and *E*_P_ is the adjusted plasma enrichment obtained by dividing venous ^13^C-Phe enrichment area under the curve between sequential biopsies with the average venous enrichment at each biopsy, as previously described [15, 16]. This adjustment reduces the potential for erroneous underestimation of the post-prandial FSR associated with the depression of plasma phenylalanine enrichment.

### Statistical analysis

The anabolic response measured with both PET/CT and stable isotope infusion was analyzed using a paired two-tail student t test with a significant difference set at *p* < 0.05. Data are presented with the average ± standard error of the mean (SEM). The relationship between baseline Ki and FSR, and between the percent increases in K_i_ and FSR, measured by PET/CT and stable isotope, respectively, was evaluated using a Pearson correlation. Statistical analyses were performed using the JMP software (Cary, NC).

Based on preliminary work from our group, we expected to be able to detect a significant, 30% increase in Ki with 80% power with a sample size of 12 subjects. Thirteen subjects were recruited for the study, and one subject dropped out of the study after the first imaging visit and was not included in any analysis. Out of the 12 subjects that completed all 3 study visits, 1 subject had invalid post-prandial *K*_*i*_ measurement, because the tissue activity curve did not saturate during the course of the imaging study and was not included in the analysis of *K*_*i*_. Furthermore, two subjects had unusable biopsy samples due to a high amount of fat and were excluded from the FSR analysis. Therefore, nine subjects had usable post-absorptive and post-prandial *K*_*i*_ and FSR data and were included in the correlation analysis.

## Results

### Baseline characteristics

**Fig. 3:**
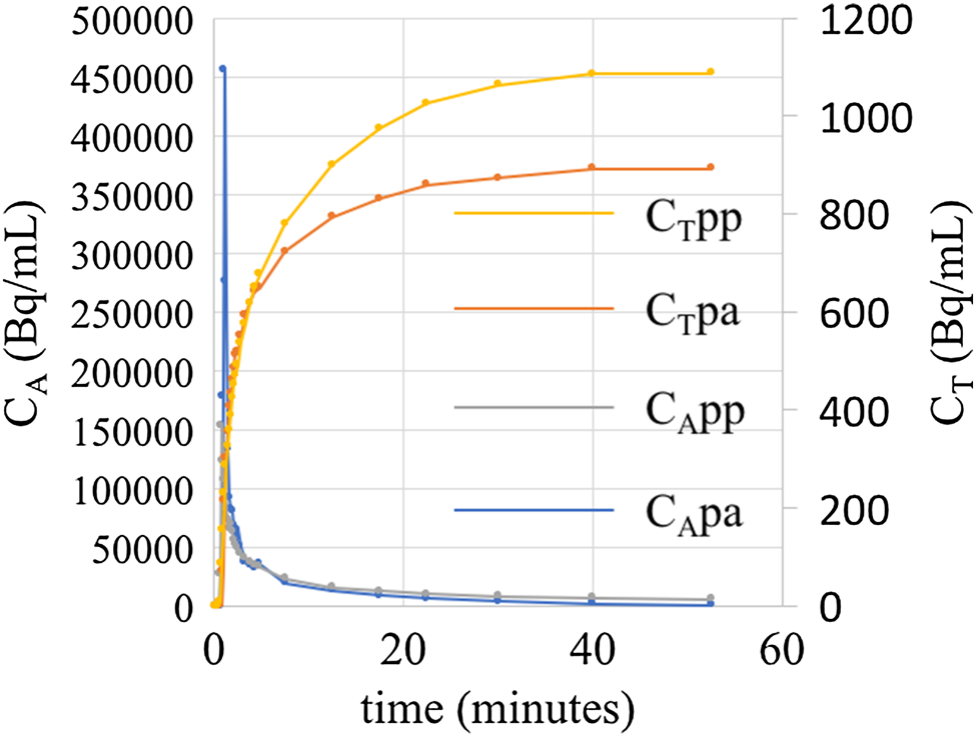
Overlay of post-absorptive and post-prandial image-derived input function IDIF (*left vertical* axis) and tissue activity curve (TAC) (*right vertical* axis) obtained for Subject 004

**Fig. 4:**
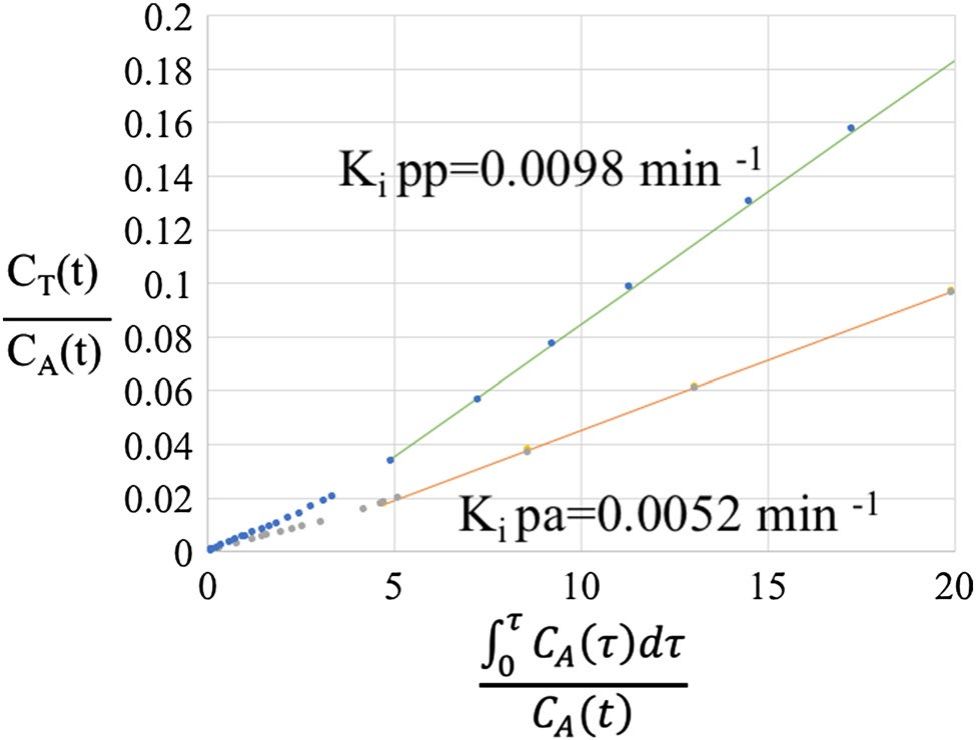
Post-absorptive and post-prandial Patlak plots for Subject 004. Linear fits to linear portions of Patlak curves are overlaid on the data

Figure 3 shows post-absorptive and post-prandial arterial input and tissue activity curves and Fig. 4 shows Patlak plots for one of the study subjects (Subject 4). Table 1 provides demographic and post-absorptive and post-prandial K_i_ and FSR values for the subjects. There was no correlation between post-absorptive *K*_*i*_ and FSR (*r* = 0.12, *p* = 0.75).

**Table 1:** Demographic data, post-absorptive (pa) *K*_*i*_ and FSR data, and post-prandial (pp) *K*_*i*_ and FSR data for the individual subjects. Blank spaces correspond to missing values where PET/CT or infusion/biopsy measurements could not be completed

Subject number	Age (years)	Weight (kg)	Height (cm)	*K*_*i*_ (pa) (min^−1^)	*K*_i_ (pp) (min^−1^)	FSR (pa) (%/h)	FSR (pp) (%/h)
1	72	73	167.6	0.0065	0.0072	0.098	0.130
2	70	72	162.6	0.0088	0.0099	0.036	0.061
3	74	86	152.4	0.0071	0.0092	0.057	0.095
4	80	52	167.6	0.0052	0.0097	0.040	0.089
5	71	62	154.9	0.0126	0.0120	0.048	0.078
6	80	44	152.4	0.0068	0.0094		
8	72	57	154.9	0.0090	0.0123	0.045	0.067
9	73	68	160.0	0.0089	0.0086	0.050	0.078
10	80	54	165.1	0.0058	0.0075	0.033	0.078
11	69	66	180.3			0.041	0.109
12	71	75	162.6	0.0074	0.0063		
13	66	73	172.7	0.0084	0.0120	0.041	0.046

Ethnic composition of cohort: 7 Caucasian, 2 Asian, 2 African-American and 1 Hispanic

### Anabolic response

**Fig. 5:**
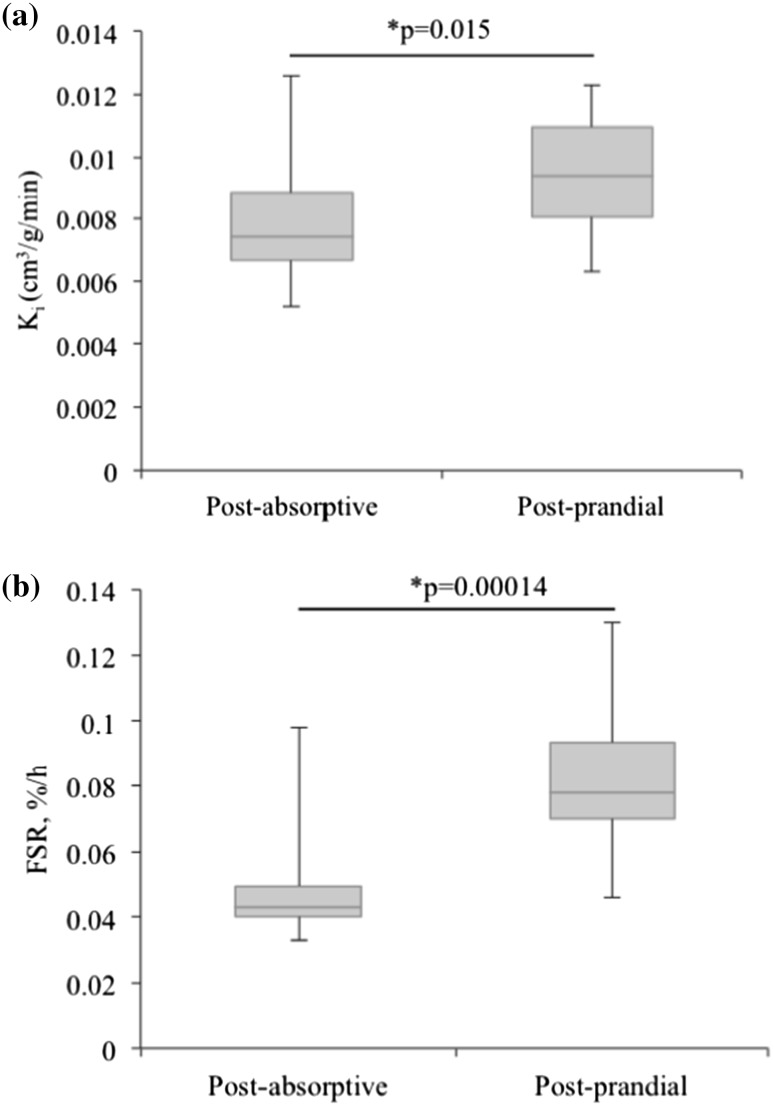
*Box* and* whiskers* plot post-absorptive and post-prandial *K*_*i*_ (*n* = 11; **a**).* Box* and* whiskers* plots of post-absorptive and post-prandial FSR (*n* = 10; **b**). For both plots, the box represents the interquartile range containing 50% of the values. The whiskers denote the maximum and minimum values (excluding outliers). The link drawn across the box is the median. Both *K*_*i*_ and FSR significantly increased in response to a 25 g whey protein supplement

**Fig. 6:**
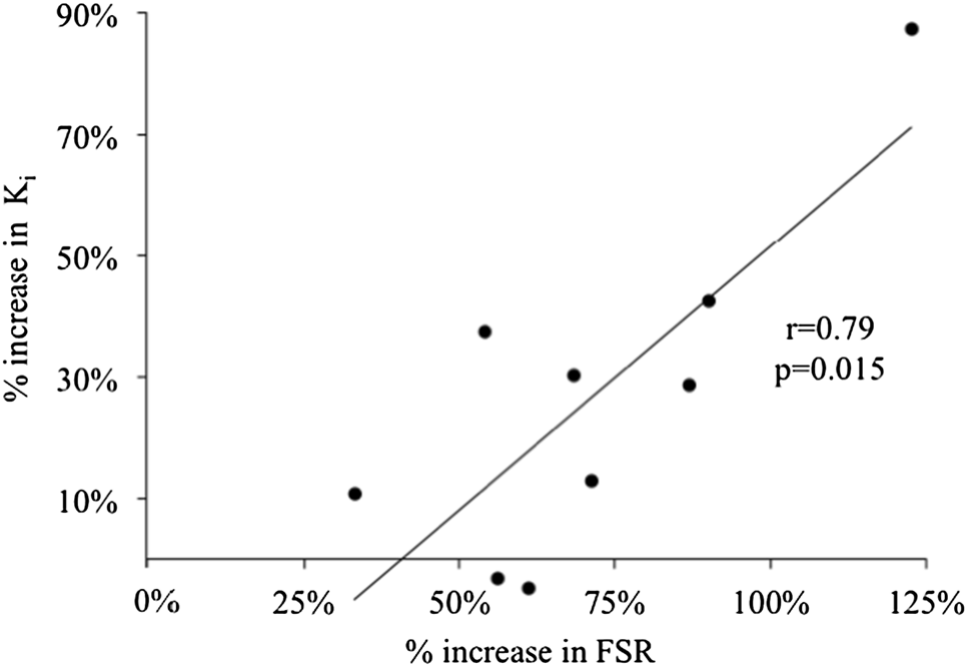
Plot of the percent increase of FSR and K_i_ in response to the 25 g whey protein supplement based on nine subjects with complete post-absorptive and post-prandial* K*_i_ and FSR measurements

A 25 g whey protein supplement elicited a 20% increase and a 70% increase in* K*_i_ (Fig. 5a) and FSR (Fig. 5b), respectively. The post-absorptive and post-prandial as well the percent increase are consistent with the previous values reported in the literature [17]. The percent increase in K_i_ and FSR in response to the whey protein supplement was significantly correlated (Fig. 6).

## Discussion

The prevalence of sarcopenia and other clinical conditions are associated with muscle-wasting motivate the development of strategies to reduce or prevent disability. The ultimate goal of such therapies is the preservation/restoration of muscle mass and functional capacity.

Therefore, the ability to quantify acute changes in skeletal muscle protein synthesis is an essential prerequisite to understanding the potential clinical/practical relevance of anabolic therapies or interventions. In this study, we demonstrated that ^11^C-MET PET/CT imaging shows an anabolic response to a clinically relevant therapeutic approach, dietary protein supplementation, and that the response is correlated to ^13^C-Phe infusion biopsy. Thus, we provided new evidence that a non-invasive quantitative imaging approach can provide valuable new information to assess the potential effects of approaches to counter muscle atrophy.

The historic gold standard for assessing skeletal muscle protein synthesis involves the infusion of a stable isotope labeled amino acid (e.g., ^13^C_6_- phenylalanine, L-[ring-^2^H_5_]phenylalanine, and L-[1-^13^C]leucine) coupled with serial muscle biopsies along with peripheral vein and occasionally femoral artery and vein blood sampling [5]. While this technique is sensitive and widely used, it has several limitations: (1) the quantity of tissue obtained by biopsy is limited (50–100 mg /muscle biopsy); (2) not all muscle beds/sites can be safely or effectively biopsied; (3) the procedure is moderately invasive, involves some discomfort, and results in a small scar; (4) obtaining suitable quality and quantity of muscle tissue from sarcopenic, frail, and/or obese individuals is challenging; (5) quantifying a change in fractional synthesis rate (FSR) requires at least two biopsies obtained over a 2–4-h period.

There are other methodologies that provide some insight into skeletal muscle response to anabolic therapies. Change in serum albumin is used as a crude clinical marker of muscle mass status [18]. Whole-body rate of appearance (Ra)/rate of disappearance (Rd) studies using stable isotopes are very useful in clinical populations, because they do not require biopsies [19]. However, the gut/splanchnic bed plays a dominant role in determining whole-body Ra/Rd and, therefore, limits the ability to determine an isolated skeletal muscle response. Deuterium-labeled water is a minimally invasive method of assessing whole-body protein turnover [20], but is not as specific as FSR, especially when measuring an acute anabolic response. Nitrogen balance studies are traditionally used for determining protein needs [21], but have limited clinical utility because of the complexity and time-intensive nature of the method.

Imaging approaches such as ^11^C-MET PET/CT imaging offer some time savings and clinical advantages. Unlike a traditional stable isotope-based infusion/biopsy process, in which two or more hours of the initial infusion are required to establish an isotopic steady state, the imaging approach requires roughly 1 h to obtain a set of image frames that can be analyzed to obtain the Patlak coefficient, which describes the forward transport and capture of the ^11^C from the blood pool into the muscle tissue bed. While other techniques such as the stable isotope pulse bolus technique are shorter than the traditional infusion protocol, it is not designed to measure the response to an anabolic stimulus [22]. In addition to time savings, PET/CT imaging is less invasive, and involves considerably less discomfort than serial skeletal muscle biopsies. Furthermore, the ^11^C-MET PET/CT approach can be carried out by a nuclear medicine technologist and does not require skilled nursing and physician support to perform the injection and imaging. Finally, the ^11^C-MET PET/CT image, as acquired in our study, provided a 3D map of ^11^C-MET uptake in a 14-cm thick volume of the thigh imaged as a stack of slices with 3.75-mm thickness. Compared to the biopsy of the vastus lateralis, this allowed us to average the K_i_ value over a large tissue volume, reducing susceptibility to tissue sampling variations. Because of this capability, PET/CT provides functional information about the general distribution of an anabolic response across an entire limb containing an array of different functional muscle groups that must operate synergistically. Our findings suggest that PET/CT imaging may function synergistically with biopsy, by focusing the use of the biopsy sample for the purposes for which it is uniquely suited, for example for studies of myofibrillar structure or of gene expression.

However, it is important to note that this approach has technical limitations as well as strengths. One limitation is that this type of study necessarily involves radiation exposure due to the administration of ^11^C-MET. In our case, the total radiation exposure associated with the two PET/CT studies was approximately 4 mSv, which is equivalent to slightly more than 1 year of background exposure. Another limitation of the approach is that in contrast to biopsy methods, it is not possible, due to the limited imaging resolution, to quantify ^11^C-MET uptake in particular compartments of the skeletal muscle tissue, such as mitochondrial or myofibrillar protein. Thus, although it offers advantages in the rapidity of acquisition and reduced invasiveness, as well as ability to survey whole muscle beds, increases in ^11^C-MET uptake represent a relatively crude measure of anabolic response of skeletal muscle tissue. In addition to technical limitations, there are also logistical and economic characteristics that should be considered. Although PET/CT systems are widely distributed throughout the United States (≅1300 systems [23]), ^11^C-MET imaging requires access to an onsite cyclotron and radiochemistry facility, and thus tends to be limited to a smaller number academic medical centers. Thus, based on this combination of advantages and limitations, we believe that at present, the primary utility of this approach is as a research tool, concentrated in academic medical centers where investigations of skeletal muscle function response are most likely to occur.

The current study is the first to show PET/CT imaging of radio-labeled amino acids such as ^11^-C-MET has an acute skeletal muscle anabolic response to administration of dietary protein and that the extent of the response is correlated to that of the ^13^C-Phe infusion/biopsy measurements, the gold standard. The previous literature has shown that ^11^C-MET uptake as measured by PET tracks with the accumulation of the 13 C labeled analog in animal necropsy models [9] and that reasonable values of PSR could be estimated in humans from PET images [8]. In a subsequent study using PET/CT, we showed that *K*_*i*_, which is highly correlated to PSR, increased in relation to exercise in a unilateral leg resistance exercise model [10]. However, that study had limited clinical relevance, because anabolic therapies in sarcopenic subjects are likely to be pharmacologic or nutritional and because that study did not show correlation to a gold standard PSR measurement, stable isotope infusion of 13 C-phenyalanine with serial biopsies. The strength of this study was that it used a nutritional intervention known to result in increased protein synthesis rate, and compared changes in image-derived kinetics of ^11^C-MET to changes in ^13^C-Phe kinetics using standard infusion/biopsy approach. We revealed that both techniques showed a significant elevation with the known anabolic stimulus and that the responses of the two approaches were correlated with statistical significance. Interestingly, despite the correlation of percentage change measures, the baseline values of *K*_*i*_ and FSR were uncorrelated. We believe that this may arise from differences in the functional roles of methionine and phenyalanine. While both are structural constituents of skeletal muscle protein, methionine is the only sulfur-containing amino acid and has important metabolic and signaling roles [24, 25] apart from its structural role. Unlike phenyalanine, methionine is not produced in the body and must be obtained from external sources. Thus, because there may be inter-individual variations in MET metabolism due to these additional metabolic and signaling roles, skeletal muscle uptake of these two amino acids may be uncorrelated in the fasted state, but be correlated in their responses to a powerful anabolic stimulus that increases incorporation of all amino-acid constituents into skeletal muscle. In summary, from our current findings, and from the previous study showing ^11^C-MET response to exercise, we believe that that this imaging method shows a similar anabolic response of skeletal muscle tissue to anabolic interventions such as nutritional therapies, exercise, and pharmacologic interventions similarly to ^13^C-Phe infusion and serial biopsy.

While our study has strengths, and the results are strongly supportive of further exploration of this approach in larger studies, there are limitations to consider. The inter-individual variability in *K*_*i*_ and in the change in *K*_*i*_ was higher than that observed for FSR. Furthermore, the correlation obtained (*r* = 0.8) was somewhat low. This is not surprising, given that the number of subjects was small, the two imaging measurements were done on different days, the imaging and biopsy studies were also done separately, and they used different compounds for the MPS measurements. Thus, our study does not establish that ^11^C-MET imaging is a surrogate measure for ^13^C-Phe infusion/biopsy study in individuals. However, we do believe that this approach demonstrates the feasibility of using the K_i_ determination from ^11^C-MET imaging as a measure of skeletal muscle anabolic response in groups of subjects, and greatly supports the use of this technique as a research tool, particularly, as described below, in subjects who may not be able to sustain a prolonged infusion/biopsy study.

We believe that this approach will have advantages as a research tool, making dynamic assessment of skeletal muscle anabolic response more accessible in the target populations expected for anabolic agents. We believe that this approach is best applied to investigations of health conditions that have a deleterious effect on PSR, such as long-term bed rest. For example, bed rest studies of healthy subjects show up to 30% decreases in PSR as measured by ^13^C-Phe infusion and biopsy. People, especially the elderly, who may under undergo 2–3 weeks of bed rest for health conditions often suffer from sarcopenia [26–28], which complicates rehabilitation. Because this approach greatly reduces the time burden and discomfort for the patient, ^11^C-MET imaging with PET/CT may be useful to characterize skeletal muscle deficits in this population. By the same token, it will also be applicable to investigate skeletal muscle function in other disease groups, such as chronic kidney disease [29–31], HIV [32, 33] or diabetic patients [34–36]. Moreover, because the PET image is co-registered with a CT image, it is possible to correlate the K_i_ measure with measures of muscle cross-sectional area and lean tissue Hounsfield Unit, a measure of fatty infiltration which is associated with altered muscle strength and physical function.

In conclusion, PET/CT with ^11^C-MET can be used to represent the aggregate response of a group of subjects to conditions that stimulate or reduce protein synthesis. From the previous work, the K_i_ responds to unilateral leg extension and flexion exercise, which stimulates protein synthesis rate as measured in biopsy/infusion studies. From this work, there is a significant positive response to whey protein ingestion, which agrees with the aggregate response in our subjects by biopsy.

## Abbreviations

FSR: Fractional synthetic rate
K_i_: Patlak Coefficient, representing fractional extraction rate Ki from plasma into a peripheral compartment (bound protein)
^13^C-Phe: L- [ring-^13^C_6_] phenylalanine
PET: Positron emission tomography
^11^C-MET: ^11^C-L-methyl-methionine
MPS: Muscle protein synthesis
PSR: Protein synthetic rate
CT: Computed tomography
IDIF: Image-derived input function
TAC: Tissue activity curve
C_A_: Activity of 11 C-MET measured in femoral artery by IDIF analysis
C_T_: Activity of 11 C-MET measured in skeletal muscle tissue at each point in the TAC
